# Enterococci in orthopedic infections: who is at risk?

**DOI:** 10.1186/2047-2994-4-S1-P70

**Published:** 2015-06-16

**Authors:** I Uckay, C Landelle, B Kressmann, A Agostinho, S Emonet, D Pittet

**Affiliations:** 1 Geneva University Hospitals, Geneva, Switzerland; 2 Infection Control Program, Geneva University Hospitals, Geneva, Switzerland

## Introduction

Orthopedic and trauma surgery is most frequently a clean surgery, unless injury-related or in the presence of spontaneous soft tissue infection. International guidelines recommend 1^st^ and 2^nd^ generation cephalosporins for perioperative prophylaxis; the later do not cover enterococci.

## Objectives

To investigate whether some patient populations/types of surgery would be particularly at risk for enterococcal infections and might benefit from an adapted prophylaxis.

## Methods

Single-center, retrospective cohort study of adult patients operated for orthopedic infections 2004-2014. Only intraoperative microbiological samples and first clinical infectious episodes were considered for analysis. We excluded recurrent infections and pediatric cases.

## Results

Among 2740 surgical interventions, enterococci were identified in 100 (3.6%) intraoperative samples. Only 33/100 (33%) infections were monomicrobial. Overall, 665 surgeries (24%) involved osteosynthesis material. Enterococcal infections were particularly related to the foot (29/429 vs. 71/2311; *p*<0.01), associated with abscesses (25/1070 vs. 75/1670; *p*<0.01), polymicrobial infections (67/572 vs. 33/1853; *p*<0.01) and underlying osteosynthesis material (35/665 vs. 55/2075; *p*<0.01). All hardware (total joint arthroplasties, plates, nails) were equally infected without predilection for a particular material. The proportion of enterococci among all pathogens in diabetic foot infections was 7%. Enterococci significantly more often responsible for diabetic foot infections (48/659 vs. 52/2081; *p*<0.01) and infections among elderly people (median age 65 years vs. 56 years, *p*<0.01). In contrast, enterococci were almost never identified in septic bursitis and native bone or joint infections. By multivariate analysis adjusting for case-mix and age, the presence of diabetic foot (odds ratio 1.9, 95%CI 1.2-2.9) and polymicrobial infection (OR 6, 95%CI 3.9-9.4) were the only variables significantly associated with enterococcal infection; while sex, age, type of material, and the exposure to antibiotic therapy prior to intraoperative sampling were not.

## Conclusion

Enterococci in orthopedic surgery are rare and mostly encountered as co-pathogens in polymicrobial infections of the ulcerating diabetic foot. There is no indication to change our antibiotic prophylaxis policy.

## Disclosure of interest

None declared.

